# Intra-Attack Vestibuloocular Reflex Changes in Ménière's Disease

**DOI:** 10.1155/2016/2427983

**Published:** 2016-11-28

**Authors:** Dario A. Yacovino, John B. Finlay

**Affiliations:** ^1^Department of Neurology, Cesar Milstein Hospital, Buenos Aires, Argentina; ^2^Memory and Balance Clinic, Buenos Aires, Argentina; ^3^Princeton University, Princeton, NJ, USA

## Abstract

Ménière's attack has been shown to temporarily alter the vestibuloocular reflex (VOR). A patient with unilateral Ménière's disease was serially evaluated with the video Head Impulse Test during single, untreated episodes of acute vertigo. Spontaneous nystagmus activity was concurrently recorded in order to establish the three typical phases of Ménière's attack (irritative, paralytic, and recovery) and correlate them with VOR performance. The onset of attack was associated with a quick change in VOR gain on the side of the affected ear. While a rapidly progressive reduction of the VOR was evident at the paralytic nystagmus phase, in the recovery phase the VOR gain returned to normal and the direction of the previous nystagmus reversed. The membrane rupture potassium intoxication theory provides a good foundation with which to explain these dynamic VOR changes and the observed triphasic direction behavior of the spontaneous nystagmus. We additionally postulated that endolymphatic fluid displacement could have a synergic effect during the earliest phase of attack.

## 1. Introduction

Ménière's disease (MD) is a fluctuating audiovestibular disorder. The recurrent vertigo attacks, among others, are the most stressful symptoms. According to the temporal direction of spontaneous nystagmus, three classic phases of the attack have been recognized: an initial “irritative” phase beating toward the affected ear, the contralateral “paralytic” phase, and the final “recovery” phase beating again toward the affected side. However, to date, few studies have considered vestibular function measurements during the progression of the attack, and the exact VOR performance in each of the three phases is unknown [1].

The new video Head Impulse Test (vHIT) provides an objective way to measure the dynamic vestibuloocular reflex (VOR) and can be used even in acute vestibular episodes [2]. This procedure is a very valuable, brief test to assess online VOR function, which is particularly helpful for studying short-lived vestibular phenomenon such as Ménière's attack.

The aim of this work is to report the VOR changes measured with vHIT throughout a single Ménière's attack in the following patient.

## 2. Case Report

**Figure 1:**
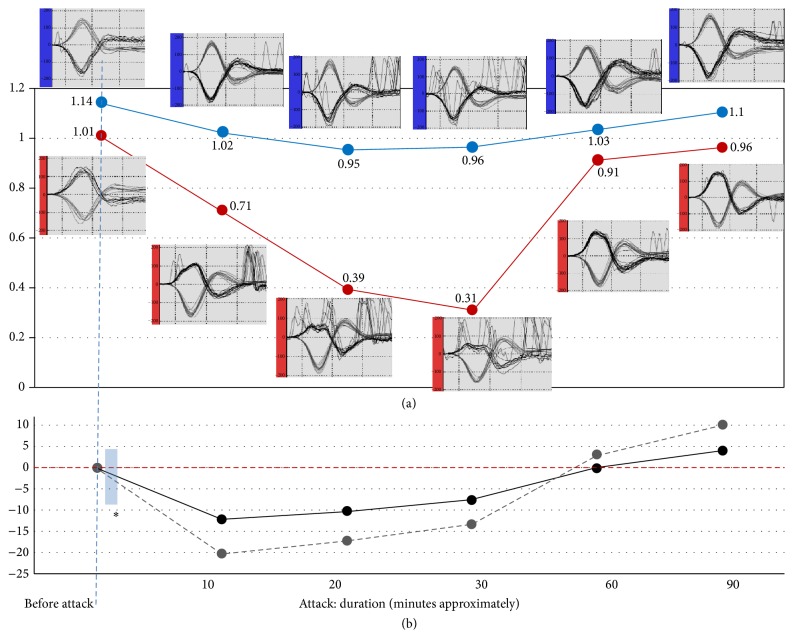
(a) The VOR regression gain values on the *y*-axis shows evolution of the right affected side (red line) and left side (blue line) during a vertigo attack from the beginning (0 minutes before attack) to the symptomatic end (90 minutes). The small plots show the video Head Impulse Test (vHIT) recordings; the velocity trajectories (°/s) of the eye (dark grey lines) and head (light grey lines) are depicted during right and left impulses. There are short time mismatches between the vHIT records and the VNG records (spontaneous nystagmus), which can be attributed to the changes and calibration of each instrument. On the right HIT, note the saturated profile of the eye velocity curve and the low VOR gain (from 1.01 to 0.31) as well as the grouped, same direction corrective saccades (at 0.71 right gain). Note also that, with the progression of the attack (at 0.39 and 0.31 right gain), the untidy saccadic movements observed on the ocular velocity baseline trace are due to the interference effect at the onset of the fast phase of the spontaneous nystagmus. At the end of the acute stage (about 60 minutes), the horizontal nystagmus changes direction, and the eye velocity curve regains its normal trajectory, although the VOR gain still shows a slight asymmetry. A week later, the gain was normal (1.09 right and 1.1 left), and no corrective saccades or spontaneous nystagmus were recorded. Technical conditions of the vHIT: number of head impulses technically accepted for analysis: from 10 to 25. Head velocity: from 140 to 190 (°/sec). (b) Spontaneous nystagmus. Velocity SPV °/sec on the *y*-axis is shown with fixation (black line) and without fixation (grey line). At 60 minutes, the nystagmus reversed to the right. ^*∗*^The irritative period was not recorded.

An 82-year-old male with right-sided MD for the last 10 years suffered spontaneous attacks of vertigo ranging in frequency from 2 to 4 times per year. He had also occasionally suffered from right posterior canal benign paroxysmal positional vertigo (PC-BPPV) in the quiescent period of the MD. He came to the clinic with typical positional brief upbeating and torsional nystagmus of right PC-BPPV on the Dix-Hallpike maneuver. As a practical routine in our clinic, a vHIT was performed prior to a Semont maneuver. Ten minutes after the maneuver, while he was in a seated position, the vertigo started again. Reexamination showed right beating horizontal nystagmus lasting 3 to 5 minutes (irritative nystagmus). After this, a change of direction was evident: progressive build-up of left beating horizontal nystagmus (paralytic nystagmus) reached a peak intensity about 10 to 15 minutes from the beginning (12°/sec of SPV with vision), accompanied by severe vertigo, nausea, sweating, and gait disequilibrium. The duration of the vertigo attack was about 1 hour. VHIT (“Eye see cam,” Interacoustics, Inc.) and spontaneous nystagmus (binocular 105 Hz Videonystagmography, VNG/V0425, Interacoustics, Inc.) measurements were taken at regular intervals throughout the episode, interchanging the goggles and recalibrating both devices at each interval (Figure 1). In order to minimize patient intolerance during the vHIT procedure, no more than twenty-five horizontal head impulses were passively and randomly applied toward each side. The horizontal VOR gains were automatically measured by the vHIT software in two forms: the instantaneous 40, 60, and 80 ms velocity gain-VOR (head and eye velocity at 40, 60, and 80 ms head movement), and the slope of the linear regression of head on eye velocity variables (*regression gain*) [3]. Since the latter measurement is a mathematically more robust value in an otherwise unstable vHIT baseline trace (i.e., generated by compensatory eye movements or spontaneous nystagmus), this method was ultimately used to document the VOR changes (Figure 1).

When the vertigo started to decrease, a recovery nystagmus (right beating) was recognized after a short quiescent period without any nystagmus. Screening tests to rule out similar conditions were done beforehand, all with normal results, including blood syphilis antibody, anti-cochlear antibody (68 KD), immunological panel, inner ear MRI, and CT Scan. New controls at 2, 7, and 30 days with BPPV still present showed normal gains on the vHIT and no spontaneous nystagmus (SN). An informed consent for the academic use of patient clinical data was obtained.

## 3. Discussion

### 3.1. Pathophysiological Discussion

Supported by the membrane rupture potassium intoxication theory, the mechanism of Ménière's attack is a dynamic, triphasic biological process (irritative, paralytic, and recovery), and the exact time frame regarding how early into the attack the VOR recordings are made is critical.

The severe reduction of the VOR during Ménière's attack has been suspected in the clinical setting. However, to the best of our knowledge, the instantaneous VOR changes over a single episode have never been well-documented.

In the intercrisis period (1 week before and 1 and 4 weeks after attack), the vHIT showed normal gain and symmetry on the 6 semicircular canals, and no corrective saccades were identified. We were unable to detect higher than normal VOR gain during the quiescent phase, as was reported in some MD patients [4]. However, there were severe reductions in VOR gain on the affected side during the attack.

Pertinent histopathologic findings in Ménière's Disease include endolymphatic hydrops and associated rupture in almost every part of the membranous labyrinth (excluding the nonampullated portions of the semicircular canals) [5]. According to this theory, the rupture of the distended membranous labyrinth would release neurotoxic potassium-rich endolymph into the perilymph. Irritative acute (depolarization phase) nystagmus, which later becomes paretic (ATPase pump blocking phase), was thought to result from a complete depolarization of either first-order vestibular afferent nerve fibers passing through the perilymph space, or of sensory cells on their synaptic area directly by the escaping endolymph, which increases perilymphatic potassium concentration [6]. This idea is supported by the observation that the direction of the spontaneous nystagmus documented during the attacks was congruent with both phases in the present case.

A group of patients in nonserial HIT studies during Ménière's attack showed variable VOR gains (from normal to reduced) [7]. Since the nystagmus during the irritative and recovery phases had the same direction (beating toward the affected ear), the authors reasoned that it was impossible to determine the phase of the attack that was studied. As shown in the present case, the attack is a dynamic process.

We detected an irritative nystagmus that was short-lived [8]. Unfortunately, we were unable to perform a vHIT at this phase since it lasted only a few minutes. It has been theorized that the presence of irritative nystagmus depends on the size of the perilymph leak into the endolymph system. A sudden and rapid increase in the potassium concentration in the perilymphatic space on the affected side would induce a paretic nystagmus without a visible irritative phase [9]. Contrarily, a smaller progressive filtration of the endolymph should induce a longer (when present) irritative phase followed by a paretic phase, which was the pattern of this case.

Using the distended membrane theory (hydrops) as a base, we postulated that the location of the membrane rupture (leaking) with respect to the ampullary cupula could induce a sudden endolymphatic fluid displacement with either ampullofugal or ampullopetal endolymphatic flow direction. In the horizontal canal, the ampullopetal is excitatory, so the irritative nystagmus should be evidently brief. On the other hand, in the case of ampullofugal flow, which is inhibitory, the induced nystagmus would have the same direction as the paralytic phase, so the initial phase should not be evident. Finally, once the paralytic phase (nerve blocking) starts the cupula deflection, no clinically visible effects should be produced.

In the case of study, the paralytic phase was associated with severe reduction of the VOR, which could not be explained by either the central adaptation or the baseline SN effect. It was recently reported that when induced in postrotatory conditions, an intense baseline SN (SPV greater than 30°/sec) could affect the VOR absolute gain value in the vHIT [10]. However, these baseline conditions were not obtained in the present case (max SPV of 12°/sec).

In the recovery nystagmus phase, the VOR returned to normal gain, compatible with central mediate adaptation nystagmus without peripheral involvement. According to the floating bias toward the side of less active vestibular input [1], when the side with the less active vestibular input recovers to levels analogous with the contralateral side, the bias becomes unmasked in the form of nystagmus toward the formerly less active side. Vestibular adaptation provides a good conceptual framework for the phenomenon observed here* in vivo* and in other similar conditions (p.e. post caloric reversal nystagmus).

### 3.2. Clinical Discussion

A causal relationship between BPPV and its subsequent development of Ménière's attack has been proposed. Studies of Ménière's disease after physical trauma have described a hypothetical mechanism in which free-floating otoliths could induce hydrops by mechanically obstructing the longitudinal flow and absorption of endolymph [11]. The particle could conceivably provoke the obstruction and accumulation of endolymphatic fluid, which would lead to membrane rupture. In our case, however, the very short period (minutes) between the Semont maneuver performed and the presented Ménière's attack makes this theoretical mechanism less plausible. As further evidence against this hypothesis, the canal reposition maneuver (CRM) was ineffective due to the fact that the Dix-Hallpike test at the follow-up remained positive. There were also various attacks of BPPV without Ménière's attack and vice versa. Another condition that could mimic the VOR features described in the presented case is the plugging of the horizontal canal due to a jamming of particles after the CRM. However, the three observed instances of direction change of the spontaneous horizontal nystagmus, the self-limited evolution, and the progressive reduction of VOR could not be explained by a single canal plugging mechanism [12]

Martinez-Lopez and coauthors reported a similar case and presented additional discussion [13]. However, there were some remarkable clinical differences. First, the ear studied had previously been submitted to a partial chemical ablation procedure (transtympanic gentamicin). Second, the nystagmus found was monophasic (same direction throughout the episode) on the horizontal axis with a vertical component soon added. However, by definition, only one of the typical three stages of attack was documented. Third, although the affected side superior canal did show a gain in VOR reduction, the horizontal did not, even when a significant horizontal component of the SN was observed. The authors hypothesized that the horizontal component could actually be of utricular origin. On the other hand, in our case the spontaneous direction-changing horizontal nystagmus throughout the single attack was associated with a rapid and severe reduction of the VOR on the same horizontal axis. Because of the severity of the vertigo attack, we attempted to minimize stimuli by only performing horizontal impulses in the vHIT. This brief study protocol allowed us to achieve a higher number of VOG and vHIT in an hour-long episode and assess the online VOR behavior during Ménière's attack. Contrary to the cochlear component (progressive hearing reduction), in this case the high VOR changes were rapid and reversible. According to other similar cases [4, 7], in the presence of a fluctuating unilateral gain in the vHIT in a recurrent vertigo case, endolymphatic hydrops should also be considered as a diagnosis.

## 4. Conclusions

This case showed a severe rapid fluctuation of the high velocity VOR during Ménière's attack. The membrane rupture, fluid displacement, and perilymphatic intoxication theories, postulated* in vitro*, properly explain the dynamic neurophysiological changes presented here, in a human case.
